# Retzius-Sparing Robot-Assisted Radical Prostatectomy: Analysis of Functional and Oncological Outcomes

**DOI:** 10.3390/medicina62091792

**Published:** 2026-09-17

**Authors:** Fırat Akdeniz, Sıtkı Ün, Uğur Boylu, Aylin Köseler

**Affiliations:** 1 Department of Urology, Faculty of Medicine, Istinye University, Istanbul 34010, Türkiye; 2 Department of Urology, Faculty of Medicine, Pamukkale University, Denizli 20160, Türkiye; sun@pau.edu.tr; 3 Department of Urology, Liv Hospital Ulus, Istanbul 34340, Türkiye; ugur@ugurboylu.com; 4 Department of Biophysics, Faculty of Medicine, Pamukkale University, Denizli 20160, Türkiye

**Keywords:** prostate cancer, robot-assisted radical prostatectomy, Retzius-sparing, urinary continence, positive surgical margin, functional outcomes, oncological outcomes, robotic surgery

## Abstract

*Background and Objectives*: Retzius-sparing robot-assisted radical prostatectomy (RS-RARP) has emerged as an alternative surgical approach designed to preserve pelvic anatomical structures involved in urinary continence. This study aimed to evaluate the perioperative, pathological, functional, and early postoperative oncological findings of RS-RARP in patients with clinically localized prostate cancer. *Materials and Methods*: This retrospective single-cohort study included 132 consecutive patients who underwent RS-RARP at a single institution between January 2017 and February 2020. Demographic, perioperative, pathological, functional, and postoperative oncological data were retrospectively collected. The primary functional outcome was postoperative urinary continence recovery. Pathological outcomes included final ISUP Grade Group and positive surgical margin status, while postoperative prostate-specific antigen (PSA) measurements and treatment-related oncological follow-up data were evaluated where available. *Results*: The median operative time was 170 min (IQR, 160–200), and the median estimated blood loss was 100 mL (IQR, 70–150). Blood transfusion was required in one patient (0.8%), and no Clavien–Dindo grade ≥ III complications were observed. Final pathological examination demonstrated ISUP Grade Group 2 disease in 64.9% of patients, and the overall positive surgical margin rate was 33.6%. The median time to both social continence (0–1 pad/day) and complete continence (0 pad/day) was 30 days. Among 130 patients with available current pad-use data, 108 (83.1%) were completely pad-free, 13 (10.0%) required one safety pad per day, and 9 (6.9%) required two or more pads per day. The median oncological follow-up was 12 months. Follow-up data were available for 131 patients; postoperative PSA measurements were available for 79 patients and last PSA measurements for 122 patients. No salvage therapy or biochemical recurrence was documented in the available clinical records. However, incomplete longitudinal PSA data precluded reliable assessment of biochemical recurrence-free survival. *Conclusions*: In this retrospective single-cohort study, RS-RARP was associated with low perioperative morbidity, acceptable pathological outcomes, and favorable early urinary continence recovery. However, the absence of a conventional RARP comparator group, the relatively short follow-up period, and incomplete longitudinal PSA and recurrence-related data preclude conclusions regarding comparative effectiveness and long-term oncological efficacy. Prospective multicenter comparative studies with standardized functional assessment, systematic PSA monitoring, and longer oncological follow-up are warranted.

## 1. Introduction

Radical prostatectomy with robot assistance has become a standard surgical approach for the treatment of prostate cancer, owing to the enhanced visualization and ergonomic advantages it offers compared with traditional surgical techniques [1,2]. The transition toward minimally invasive approaches has been accompanied by improvements not only in perioperative outcomes but also in functional recovery. In particular, the Retzius-sparing approach has been reported to facilitate earlier recovery of urinary continence by preserving the anatomical integrity of the anterior pelvic supporting structures [3]. Nevertheless, further evaluation of perioperative, functional, and oncological outcomes in well-characterized RS-RARP cohorts is needed to better define the clinical performance and safety of this surgical approach.

In comparative surgical studies, differences in baseline patient characteristics may influence the observed functional and oncological outcomes, highlighting the importance of accounting for potential selection bias when interpreting comparisons between surgical approaches [4]. The use of robot-assisted radical prostatectomy has evolved significantly from the traditional anterior technique to approaches that preserve the Retzius space [5]. These technical modifications reflect a strategic focus on improving early postoperative urinary continence [6,7]. The Retzius-sparing approach primarily aims to reduce surgical morbidity and accelerate functional recovery by preserving the bladder neck support mechanisms and periurethral structures [8,9]. Previous comparative and retrospective studies have emphasized that differences in baseline patient characteristics and clinical heterogeneity should be considered when interpreting functional and oncological outcomes across surgical approaches [10,11,12]. Comparative studies have demonstrated that the Retzius-sparing approach, through preservation of pelvic supportive anatomy, may facilitate earlier recovery of urinary continence compared with conventional approaches [13,14].

On the other hand, to maintain oncological control, the influence of risk factors such as clinical T3 stage on surgical outcomes should be thoroughly evaluated in large-scale, multicenter cohorts [15,16]. In addition, surgeon experience and differences in surgical training should be considered important factors that may influence the outcomes of robot-assisted surgery [17]. The existing evidence suggests that conventional and Retzius-sparing approaches provide comparable oncological outcomes, particularly with regard to positive surgical margin rates [18]. However, the Retzius-sparing technique may provide functional benefits in terms of postoperative urinary continence by preserving the anatomical structures supporting the bladder and urethra [19]. Preservation of these pelvic supportive structures may facilitate earlier recovery of urinary continence [20]. Nevertheless, when interpreting outcomes from retrospective analyses of different surgical approaches, potential selection bias and differences in baseline patient characteristics should be carefully considered [21]. Selection bias and residual confounding are inherent concerns in retrospective studies and should therefore be acknowledged when interpreting surgical outcomes and the methodological limitations of such studies [22].

Therefore, the present study was designed as a retrospective single-cohort analysis of patients who underwent Retzius-sparing robot-assisted radical prostatectomy (RS-RARP). The primary objective was to evaluate perioperative and functional outcomes, with particular emphasis on postoperative urinary continence recovery. Pathological and early oncological outcomes, including positive surgical margin status and postoperative prostate-specific antigen levels, were also assessed. By providing a comprehensive evaluation of a consecutive institutional RS-RARP cohort, this study aimed to characterize the functional and oncological outcomes of the technique in routine clinical practice.

## 2. Materials and Methods

### 2.1. Study Design and Patient Population

**Figure 1:**
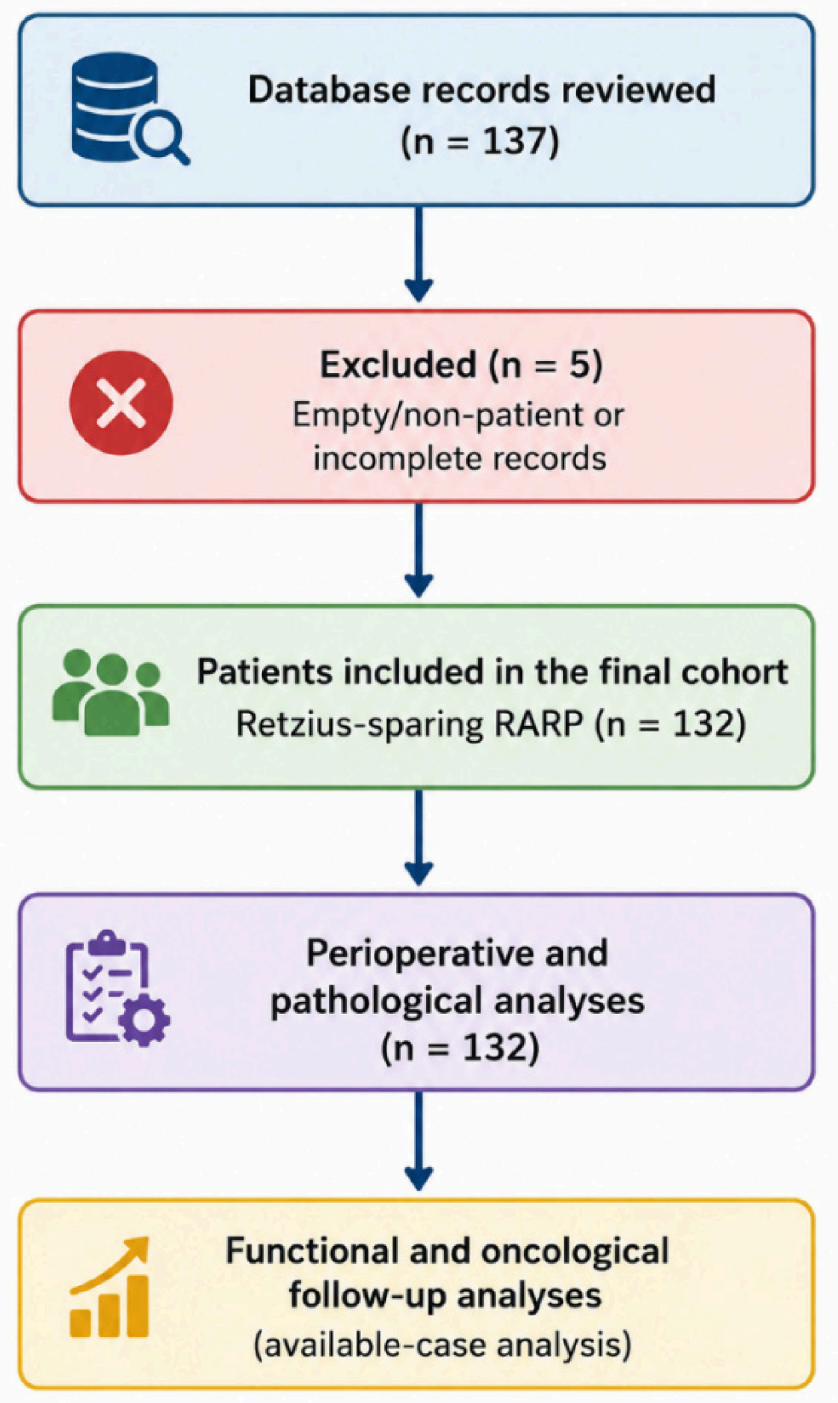
Flowchart illustrates patient selection and study inclusion. A total of 137 database records were reviewed. After exclusion of five incomplete or non-patient records, 132 patients who underwent Retzius-sparing robot-assisted radical prostatectomy (RS-RARP) were included in the final analysis.

This retrospective cohort study included patients with clinically localized prostate cancer who underwent Retzius-sparing robot-assisted radical prostatectomy (RS-RARP) at our institution between 10 January 2017 and 19 February 2020. During the study period, a total of 132 radical prostatectomies were performed at our institution, all of which were performed using the Retzius-sparing robot-assisted approach. No open, laparoscopic, or conventional robot-assisted radical prostatectomies were performed during this period. A total of 137 database records were reviewed. After exclusion of five incomplete or non-patient records, 132 patients who underwent RS-RARP were included in the final study cohort (Figure 1). All patients included in this cohort underwent RS-RARP; therefore, no patient-specific selection or allocation criteria were applied to choose between RS-RARP and conventional RARP. Patients with available preoperative clinical data, perioperative records, pathological findings, and postoperative follow-up information were included in the analysis. Outcomes with missing follow-up data were evaluated using an available-case approach. All included patients underwent RS-RARP using a posterior transperitoneal approach through the Douglas pouch, without entering the Retzius space.

### 2.2. Data Collection

Patient demographic, clinical, perioperative, pathological, functional, and oncological follow-up data were retrospectively collected from the electronic medical record system and the institutional database. Demographic variables included age, body mass index (BMI), and smoking status. Comorbidities assessed included diabetes mellitus, hypertension, coronary artery disease, the Charlson Comorbidity Index (CCI), and the American Society of Anesthesiologists (ASA) physical status classification. Urological history included previous abdominal surgery, prior surgery for benign prostatic hyperplasia (BPH), and the use of medical therapy for BPH.

Preoperative oncological evaluation included serum prostate-specific antigen (PSA) level, prostate volume, biopsy International Society of Urological Pathology (ISUP) Grade Group, number of positive biopsy cores, multiparametric magnetic resonance imaging (mpMRI) findings, Prostate Imaging Reporting and Data System (PI-RADS) score, presence of extracapsular extension, clinical T stage, and European Association of Urology (EAU) risk classification.

Perioperative variables included operative time, console time, estimated blood loss, performance of pelvic lymph node dissection, nerve-sparing surgery, bladder neck preservation, intraoperative complications, requirement for blood transfusion, and drain placement. Pathological assessment included prostate specimen weight, pathological ISUP Grade Group, pathological T and *N* stages, surgical margin status, seminal vesicle invasion, capsular invasion, lymph node involvement, bladder neck invasion, and perineural invasion. Both preoperative prostate biopsy specimens and final radical prostatectomy specimens were evaluated by pathologists at the same institution.

Functional outcomes included pad use during the first postoperative week and at subsequent follow-up visits after catheter removal, time to urinary continence recovery, current continence status, erectile function, and participation in erectile rehabilitation. Oncological follow-up included postoperative PSA measurements, the most recent PSA value at the last follow-up, and the administration of adjuvant or salvage therapy. Documented biochemical recurrence and time-to-recurrence data were reviewed from the available postoperative records. However, because longitudinal recurrence-related data were not systematically recorded for all patients, formal biochemical recurrence-free survival and time-to-recurrence analyses were not performed.

### 2.3. Surgical Technique

Retzius-sparing robot-assisted radical prostatectomy (RS-RARP) was performed using a posterior transperitoneal approach through the Douglas pouch, avoiding entry into the Retzius space. During the procedure, the puboprostatic ligaments, endopelvic fascia, dorsal venous complex, and anterior supportive structures of the bladder were preserved whenever anatomically and oncologically feasible. The procedure was completed in accordance with established Retzius-sparing surgical principles. All procedures were performed using the same robotic surgical platform by surgeons experienced in robot-assisted radical prostatectomy.

### 2.4. Study Outcomes

The primary functional outcome of the study was postoperative urinary continence recovery. Continence was defined as the use of no pads or a single safety pad per day. Secondary functional outcomes included time to continence recovery, daily pad use, erectile function, and the requirement for postoperative erectile rehabilitation.

The oncological outcomes included positive surgical margin status, pathological stage, lymph node status, and postoperative prostate-specific antigen (PSA) levels. Biochemical recurrence was predefined as a serum PSA level of ≥0.2 ng/mL confirmed on two consecutive measurements. Documented biochemical recurrence was assessed from the available postoperative records. However, because longitudinal recurrence-related data were not systematically recorded for all patients, formal biochemical recurrence-free survival and time-to-recurrence analyses were not performed.

### 2.5. Statistical Analysis

The normality of continuous variables was assessed using the Shapiro–Wilk test. Normally distributed continuous variables are presented as mean ± standard deviation (SD), whereas non-normally distributed variables are presented as median with interquartile range (IQR), as appropriate. Categorical variables are presented as frequencies and percentages. Time to urinary continence recovery was summarized using time-to-event analysis, with patients who had not achieved the specified continence endpoint at the last available follow-up treated as censored observations. Spearman correlation analysis was used to explore associations among selected demographic, perioperative, pathological, and functional variables. All statistical analyses were two-sided, and a *p* value of <0.05 was considered statistically significant where inferential analyses were performed.

## 3. Results

A total of 132 patients who underwent Retzius-sparing robot-assisted radical prostatectomy (RS-RARP) were included in the study. The median age was 62.0 years (interquartile range [IQR], 57.0–66.0), the median body mass index (BMI) was 26.4 kg/m^2^ (IQR, 24.9–28.1), and the median Charlson Comorbidity Index (CCI) was 1.0 (IQR, 0.0–1.2). Diabetes mellitus was present in 15 patients (11.4%), hypertension in 50 (37.9%), and coronary artery disease in 19 (14.4%). According to the American Society of Anesthesiologists (ASA) physical status classification, 102 patients (77.3%) were classified as ASA I, 22 (16.7%) as ASA II, and 8 (6.1%) as ASA III.

The median preoperative serum prostate-specific antigen (PSA) level was 6.45 ng/mL (IQR, 4.72–10.15), and the median number of positive biopsy cores was 3.5 (IQR, 2.0–5.0). Based on preoperative biopsy pathology, 70 patients (53.0%) had International Society of Urological Pathology (ISUP) Grade Group 1 disease, 46 (34.8%) Grade Group 2, 11 (8.3%) Grade Group 3, 3 (2.3%) Grade Group 4, and 2 (1.5%) Grade Group 5. According to the EAU risk classification recorded in the study database, 59 patients (44.7%) were categorized as low risk, 60 (45.5%) as intermediate risk, and 13 (9.8%) as high risk. The baseline demographic, clinical, and preoperative oncological characteristics of the study cohort are summarized in Table 1.

Perioperative outcomes are summarized in Table 2. The median skin-to-skin operative time was 170 min (IQR, 160–200), while the median console time dedicated to prostatectomy was 100 min (IQR, 90–112.5). The median total console time was 107.5 min (IQR, 90–120), and the median estimated blood loss was 100 mL (IQR, 70–150). Urinary catheter removal was performed at a median of 12 days postoperatively (IQR, 10–14). Perioperative complications occurred in 4 patients (3.0%), with no major complications (Clavien–Dindo grade ≥ III) observed. Blood transfusion was required in one patient (0.8%). Pelvic lymph node dissection was performed in 23 patients (17.4%). Among these patients, the median number of lymph nodes removed was 13 (IQR, 10–16).

Pathological outcomes are summarized in Table 3. The median prostate specimen weight was 52.0 g (IQR, 43.0–60.0). Final pathological ISUP Grade Group data were available for 131 patients. Of these, 18 (13.7%) had Grade Group 1 disease, 85 (64.9%) Grade Group 2, 19 (14.5%) Grade Group 3, 4 (3.1%) Grade Group 4, and 5 (3.8%) Grade Group 5. Pathological T stage was also available for 131 patients: 59 (45.0%) had pT2 disease, 50 (38.2%) had pT3a disease, and 22 (16.8%) had pT3b disease. Positive surgical margins were identified in 44 patients (33.6%). Capsular involvement was present in 70 patients (53.4%), seminal vesicle invasion in 17 (13.1%), and microscopic bladder neck invasion in 4 (3.1%).

Among the 70 patients with biopsy ISUP Grade Group 1 disease, 18 remained ISUP Grade Group 1 on final pathological examination, whereas 51 patients (72.9%) were upgraded to ISUP Grade Group ≥ 2 following radical prostatectomy. Final pathological Grade Group data were unavailable for one patient in this subgroup. The upgraded cases represented 38.6% of the entire study cohort.

**Figure 2:**
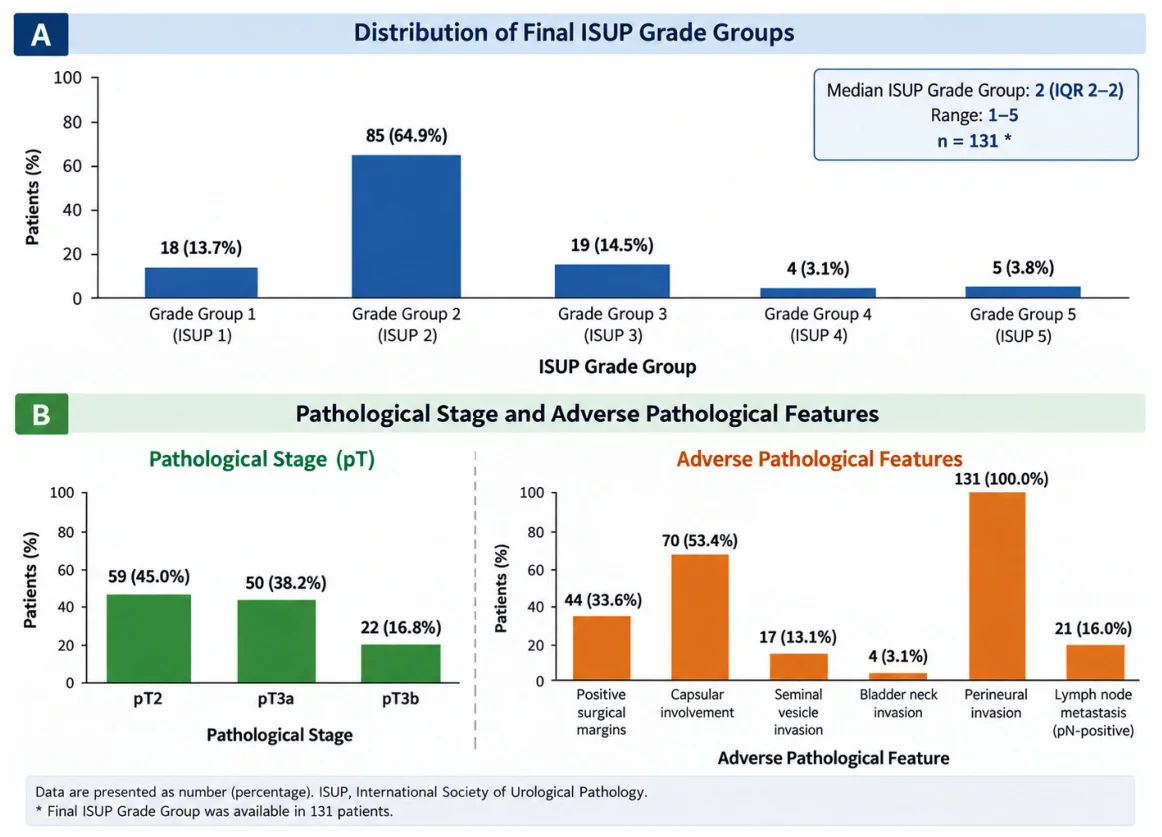
Distribution of pathological findings following Retzius-sparing robot-assisted radical prostatectomy. (**A**) Distribution of final ISUP Grade Groups. (**B**) Distribution of pathological stage (pT) and adverse pathological features, including positive surgical margins, extraprostatic extension, seminal vesicle invasion, lymphovascular invasion, and perineural invasion. Data are presented as number (percentage).

The distribution of final ISUP Grade Groups, pathological stage, and adverse pathological features, including positive surgical margins, extraprostatic extension, seminal vesicle invasion, lymphovascular invasion, and perineural invasion, is presented in Figure 2.

Functional outcomes are summarized in Table 4. The median time to social continence (0–1 pad/day) was 30 days (IQR, 15–56.3), while the median time to complete continence (0 pad/day) was 30 days (IQR, 15–90). Among 130 patients with available current pad-use data, 108 (83.1%) were completely pad-free, 13 (10.0%) required one pad per day, and 9 (6.9%) required two or more pads per day. Postoperative erectile rehabilitation was performed in 117 of 131 patients with available data (89.3%).

**Figure 3:**
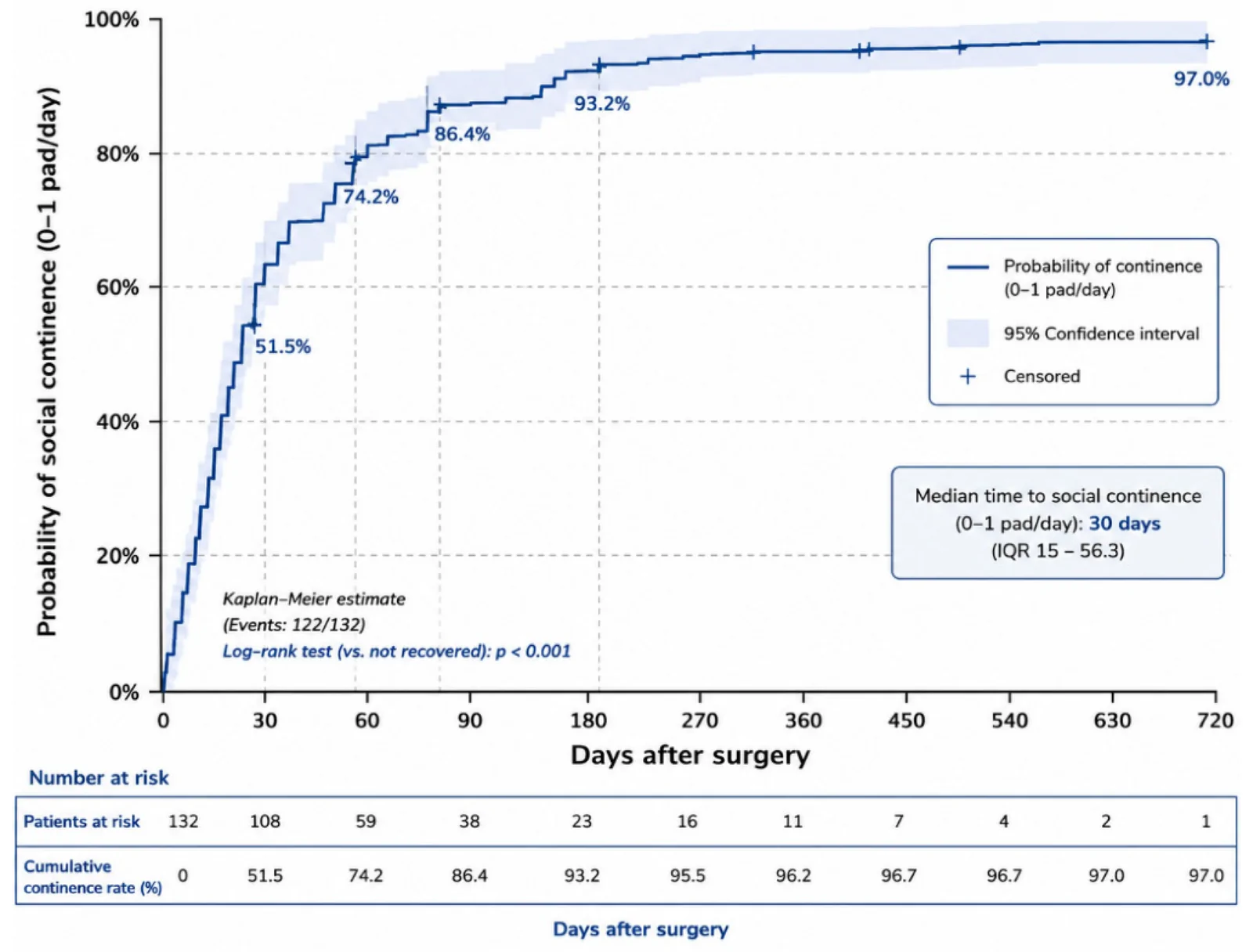
Kaplan–Meier curve showing cumulative recovery of social continence (0–1 pad/day) after Retzius-sparing robot-assisted radical prostatectomy. Shaded areas represent the 95% confidence interval, and tick marks indicate censored observations.

Data are presented as median (IQR) or *n* (%). Percentages were calculated using the number of patients with available data for each variable. Current pad use and continence status were available for 130 patients, whereas erectile rehabilitation and current erectile function data were available for 131 patients (Figure 3).

**Figure 4:**
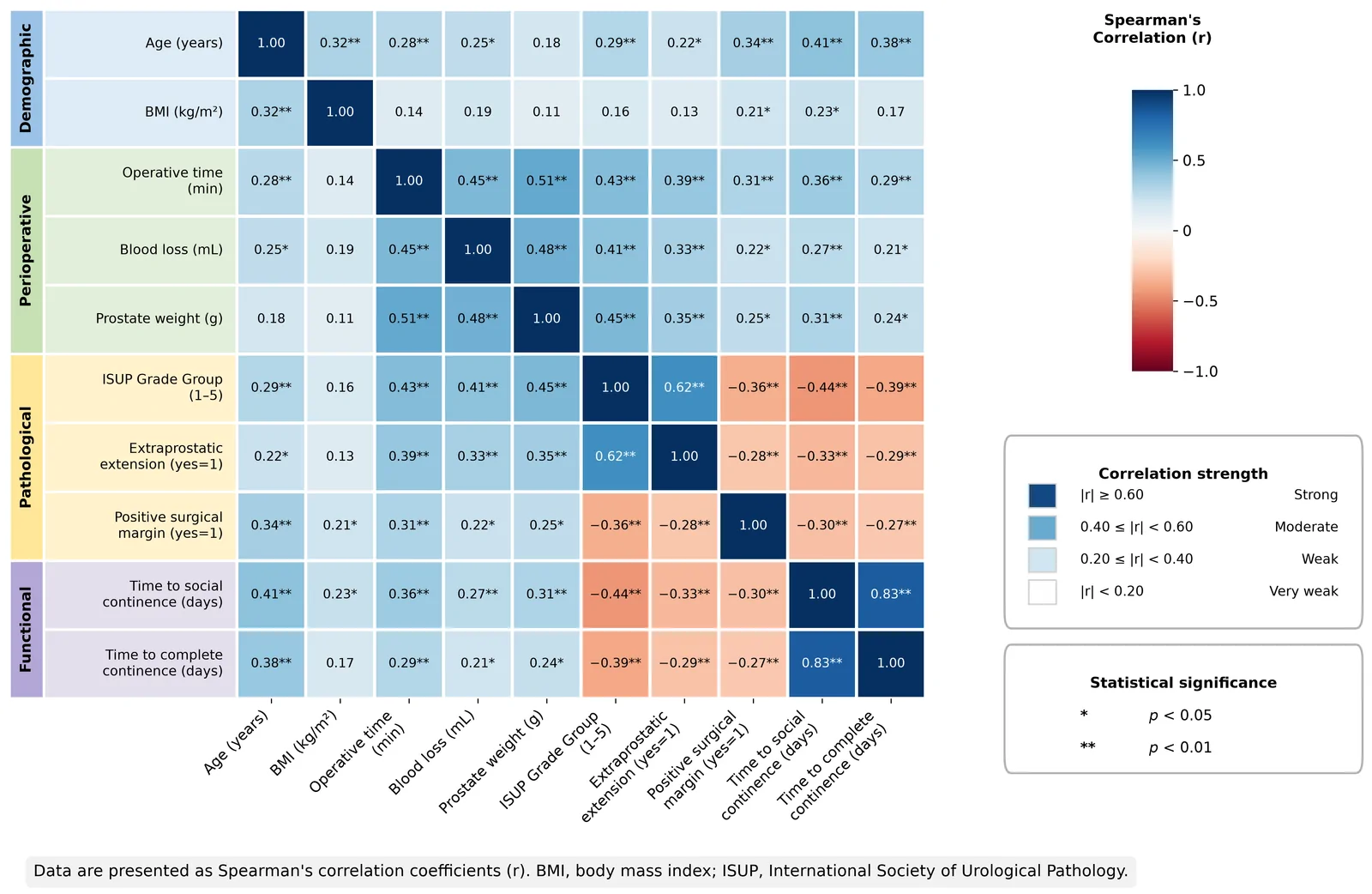
Spearman correlation heatmap illustrating the relationships among demographic, perioperative, pathological, and functional variables. Positive correlations are shown in blue and negative correlations in red. Color intensity reflects the strength of the correlation coefficient (r).

To further explore the relationships among demographic, perioperative, pathological, and functional variables, a correlation analysis was performed (Figure 4). Moderate positive correlations were observed between operative time and estimated blood loss, whereas adverse pathological features showed inverse correlations with postoperative continence recovery. Functional variables, particularly time to social continence and time to complete continence, demonstrated the strongest positive association.

**Figure 5:**
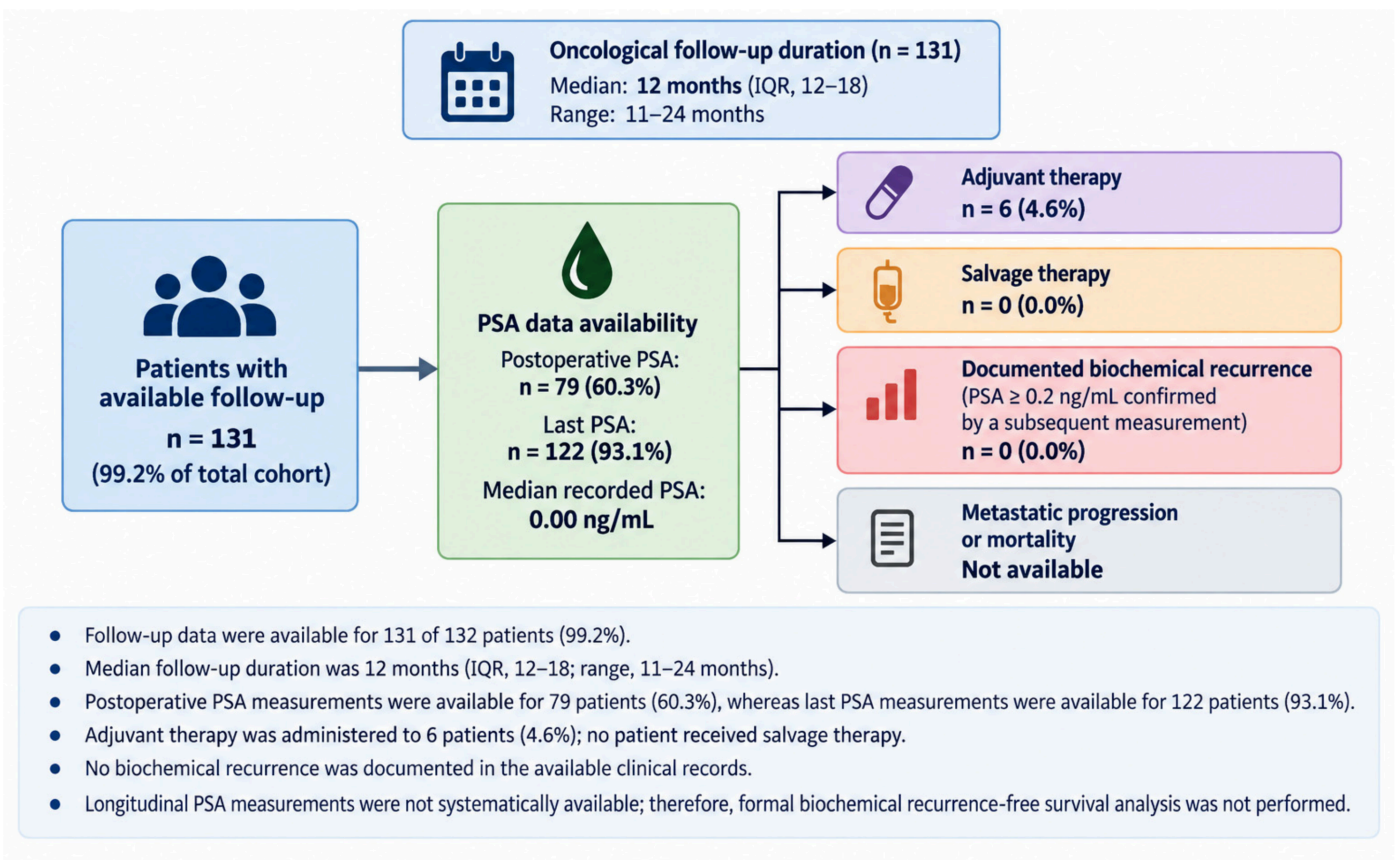
Postoperative oncological follow-up after Retzius-sparing robot-assisted radical prostatectomy. Follow-up data were available for 131 patients. Postoperative PSA and last PSA measurements were available for 79 and 122 patients, respectively. Six patients received adjuvant therapy, whereas no salvage treatment or documented biochemical recurrence was identified during the available follow-up. Because longitudinal PSA measurements were incomplete, formal biochemical recurrence-free survival analysis was not performed.

Oncological outcomes are summarized in Table 5 and Figure 5. Postoperative PSA and oncological follow-up data were available for 131 patients. The median oncological follow-up duration was 12.0 months (IQR, 12.0–18.0; range, 11–24 months). The median first postoperative PSA level was 0.00 ng/mL (IQR, 0.00–0.014), and the median last PSA level was 0.00 ng/mL (IQR, 0.00–0.00). At the last documented assessment, all 131 patients had a PSA level < 0.2 ng/mL. Adjuvant therapy was administered to 6 patients (4.6%), whereas no patient received salvage therapy. No documented biochemical recurrence was identified during the available follow-up period. Data regarding metastatic progression and mortality were not consistently available in the retrospective database.

Early oncological follow-up data are summarized in Table 5. Follow-up data were available for 131 patients, with a median follow-up duration of 12 months (IQR, 12–18). Postoperative PSA measurements were available for 79 patients, and last PSA measurements were available for 122 patients; the median PSA value was 0.00 ng/mL (IQR, 0.00–0.00) at both assessments. Adjuvant treatment was administered to 6 of 131 patients (4.6%), whereas no patient received salvage treatment. No biochemical recurrence was documented in the available clinical records. However, because longitudinal PSA measurements and recurrence-related data were not systematically available for all patients, formal biochemical recurrence-free survival and time-to-recurrence analyses were not performed.

## 4. Discussion

In this retrospective single-cohort study, we comprehensively evaluated the perioperative, pathological, functional, and early postoperative oncological outcomes of patients undergoing Retzius-sparing robot-assisted radical prostatectomy (RS-RARP). Several findings merit consideration. First, RS-RARP was associated with low estimated blood loss, a very low transfusion rate, and a low incidence of perioperative complications in this cohort [8,14]. Second, the positive surgical margin rate was within the range reported in contemporary RS-RARP series, although the absence of a comparator group precludes conclusions regarding comparative oncological efficacy [14,18]. Third, early functional outcomes were favorable, with a median time to both social and complete continence of 30 days and a high proportion of patients achieving pad-free continence during follow-up [6,7,19,20]. Finally, no biochemical recurrence was documented during the available follow-up period. However, postoperative PSA measurements were available for 79 patients and last PSA measurements for 122 patients, indicating incomplete longitudinal PSA data. Together with the relatively short follow-up period, this limits the assessment of biochemical recurrence and precludes conclusions regarding biochemical recurrence-free survival or long-term oncological efficacy.

RS-RARP, first described by Galfano and colleagues in 2010, represents an important evolution in robotic prostate surgery [23,24]. In the conventional anterior approach, dissection through the Retzius space involves dissection of or around key anatomical structures, including the puboprostatic ligaments, endopelvic fascia, dorsal venous complex, and anterior supportive structures of the bladder [23,25]. In contrast, RS-RARP accesses the prostate through a posterior route via the Douglas pouch, allowing these structures to remain largely preserved. Preservation of the puboprostatic ligaments, endopelvic fascia, Santorini venous plexus, and related periurethral support structures may help maintain bladder neck position and membranous urethral length, both of which are considered important determinants of early postoperative continence recovery [26,27,28,29]. These anatomical characteristics provide a plausible mechanistic basis for the favorable early continence outcomes reported after RS-RARP and support its role as a function-preserving surgical approach in appropriately selected patients [23,24].

The perioperative outcomes observed in the present study are consistent with those reported in previously published RS-RARP series. The median operative time was 170 min (IQR, 160–200), while the median console time was 100 min for the prostatectomy phase and 107.5 min overall. Previous RS-RARP series have reported median operative times ranging from 162 to 214 min and console times from 96 to 214 min [23,28,30,31]. The median estimated blood loss was 100 mL in our cohort, which is within the 100–200 mL range reported in previous multicenter studies [30,32,33]. Only one patient (0.8%) required blood transfusion, and the overall perioperative complication rate was 3.0%, with no major complications (Clavien–Dindo grade ≥ III). Overall, these findings indicate an acceptable perioperative profile in the present cohort; however, the absence of a comparator group precludes conclusions regarding the relative perioperative safety of RS-RARP compared with conventional RARP.

RS-RARP has been reported to be technically more demanding than conventional RARP, particularly during the early phase of the learning curve [23,24]. The confined operative field during posterior dissection, lack of clear lateral anatomical landmarks for pedicle dissection, and close proximity of the ureters may contribute to the technical complexity of the procedure [23,34]. Nevertheless, learning-curve analyses have demonstrated progressive improvements in operative time, intraoperative blood loss, and perioperative outcomes with increasing surgical experience. Previous studies have suggested that experienced robotic surgeons may achieve greater procedural proficiency after approximately 50–90 cases, with improvements also reported in margin and functional outcomes during the learning process [30,34,35]. The low complication and transfusion rates observed in the present cohort are consistent with previous reports supporting the feasibility of RS-RARP when performed by surgeons experienced in robotic prostate surgery [23,24,30].

Functional recovery represents one of the notable findings of the present study. Among the 130 patients with available current pad-use data, 108 (83.1%) were completely pad-free, 13 (10.0%) required one safety pad per day, and 9 (6.9%) required two or more pads per day at the last follow-up. The median time to social continence was 30 days (IQR, 15–56.3), and the median time to complete continence was also 30 days (IQR, 15–90). These findings indicate favorable early postoperative continence recovery within the present RS-RARP cohort and are consistent with the early functional outcomes reported in previous RS-RARP series [23,28].

The functional rationale for RS-RARP is closely related to preservation of the anatomical support system surrounding the urethra. The puboprostatic ligaments, endopelvic fascia, and Santorini venous plexus constitute important anterior stabilizing structures, while the periurethral connective tissues, levator ani muscles, and bladder neck collectively contribute to the maintenance of continence [23,25]. Chang et al. demonstrated less disruption of key pelvic support structures, including the levator ani muscles, pubourethral ligaments, and puboprostatic ligaments, with the Retzius-sparing approach [28]. Preservation of these structures may limit bladder descent and urethral hypermobility. Dynamic magnetic resonance imaging studies have additionally demonstrated preservation of a higher anterior bladder position after RS-RARP, which may facilitate posterior compression of the membranous urethra during increases in intra-abdominal pressure [19,20]. Together, these observations provide a plausible anatomical explanation for early continence recovery after RS-RARP.

Comparative clinical evidence also supports a potential early continence advantage of the Retzius-sparing approach. Previous studies have reported lower urinary incontinence rates at hospital discharge after RS-RARP than after the conventional approach (0.046 vs. 0.357), with continence rates after RS-RARP reaching 80%, 92%, and 96% at 1, 3, and 6 months, respectively [29,33]. In the randomized controlled trial by Dalela, Menon, and colleagues, 0-pad continence rates were higher in the RS-RARP group than in the conventional RARP group at early postoperative time points, including postoperative day 1 (42% vs. 15%), 1 month (68% vs. 33%), 3 months (76% vs. 60%), and 6 months (93% vs. 73%) [36]. Other randomized studies have similarly reported more rapid early continence recovery after RS-RARP [23,37]. These comparative data provide important context for the functional outcomes observed in our cohort but should not be interpreted as a direct comparison within the present study.

These findings are further supported by systematic reviews and meta-analyses. The Cochrane systematic review reported better urinary continence outcomes with the Retzius-sparing approach during the first six postoperative months compared with standard robot-assisted laparoscopic prostatectomy, although differences diminished by 12 months [38]. Similarly, subsequent systematic reviews and meta-analyses have suggested that the principal functional advantage of RS-RARP lies primarily in faster early continence recovery rather than a substantial long-term difference in continence rates [6,32,38,39,40,41]. The findings of the present cohort are consistent with this pattern. Nevertheless, because our study did not include a conventional RARP comparator group, the observed early continence recovery cannot be interpreted as evidence of superiority of RS-RARP over other surgical approaches.

From a pathological perspective, 64.9% of patients with available final pathological grading had ISUP Grade Group 2 disease, whereas 21.4% had Grade Group ≥ 3 disease. The overall positive surgical margin rate was 33.6%, which is within the range reported in previous RS-RARP series [14,26,31]. Fonseca et al. reported an overall positive surgical margin rate of 33% in a cohort of 208 patients, with rates of 28%, 39%, and 44% for pT2, pT3a, and pT3b disease, respectively [26]. Galfano et al. reported an overall positive surgical margin rate of 31%, including 14.1% for pT2 disease and 42.8% for pT3–4 disease [14]. These comparisons place the positive surgical margin rate observed in our cohort within the range of previously published RS-RARP experience; however, differences in pathological stage, patient selection, tumor characteristics, and surgical experience should be considered when comparing studies.

Concerns have historically been raised that the restricted posterior operative field and technical complexity of RS-RARP might compromise oncological radicality, particularly during the learning phase [23,24]. More recent comparative studies and meta-analyses have generally not demonstrated a clear difference in overall positive surgical margin rates between RS-RARP and conventional RARP. Chung et al., in a systematic review and meta-analysis including 12 studies and 2673 patients, reported no significant difference in overall positive surgical margin rates between the two approaches (OR 0.88, 95% CI 0.72–1.06; *p* = 0.20) [36]. Similarly, Jiang et al. found no significant difference in positive surgical margin rates (OR 1.40, 95% CI 0.88–2.33; I^2^ = 6%) [32], and the Cochrane review found no convincing evidence of a difference in margin positivity between the two techniques [38]. These data suggest that the early functional advantages reported with RS-RARP do not necessarily occur at the expense of surgical margin outcomes, although pathological stage, tumor location, surgeon experience, and patient selection remain important considerations.

The pathological findings of the present study should therefore be interpreted descriptively. Capsular involvement and seminal vesicle invasion were observed in 53.4% and 13.1% of patients, respectively, and the overall positive surgical margin rate was 33.6%. Because the present study did not include a conventional RARP comparator group, no conclusions can be drawn regarding comparative oncological efficacy or oncological non-inferiority. The favorable early continence recovery observed in our cohort should likewise be interpreted independently from comparative oncological claims.

Postoperative PSA data provide only limited information regarding early oncological status in this cohort. Follow-up data were available for 131 patients; however, postoperative PSA measurements were available for 79 patients and last PSA measurements for 122 patients. Among the recorded measurements, the median PSA value was 0.00 ng/mL (IQR, 0.00–0.00). No salvage therapy was recorded, and no biochemical recurrence was documented in the available clinical records. Nevertheless, the incomplete availability of longitudinal PSA measurements and the median oncological follow-up of only 12 months substantially limit assessment of recurrence. Accordingly, these observations should not be interpreted as evidence of biochemical recurrence-free survival or definitive oncological efficacy. Studies with longer follow-up have shown that biochemical recurrence may occur several years after radical prostatectomy [42,43,44]. Prospective studies with systematic longitudinal PSA monitoring and longer follow-up are therefore required to characterize biochemical recurrence-free, metastasis-free, cancer-specific, and overall survival after RS-RARP.

Emerging technologies may further refine the balance between functional preservation and oncological control in robotic prostate surgery. Intraoperative approaches such as NeuroSAFE may facilitate nerve-sparing while allowing real-time assessment of surgical margins [45,46,47,48], whereas PSMA-targeted imaging and fluorescence-guided techniques may improve intraoperative tumor localization and surgical precision [49,50,51,52]. Although these technologies remain under investigation, their future integration with function-preserving approaches such as RS-RARP warrants prospective evaluation.

Several limitations should be acknowledged. First, this was a retrospective, single-center, single-cohort study without a conventional RARP comparator group, which limits causal inference and prevents direct assessment of the relative efficacy or safety of RS-RARP. Second, the sample size was relatively modest, and the median oncological follow-up of 12 months was insufficient to assess long-term oncological outcomes. Although no biochemical recurrence was documented in the available clinical records, longitudinal PSA measurements were incomplete, with postoperative PSA data available for 79 patients and last PSA data for 122 patients. Therefore, formal biochemical recurrence-free survival and time-to-recurrence analyses could not be performed. Data regarding metastatic progression, cancer-specific survival, and overall survival were also not consistently available. Third, functional outcomes were obtained retrospectively from routine clinical records rather than prospectively collected validated patient-reported outcome measures, which may have introduced measurement and reporting bias. Furthermore, continence and erectile function data were not available for every patient, requiring available-case analyses for these outcomes.

An additional limitation concerns treatment selection among patients with low-risk disease. The cohort underwent surgery between 2017 and 2020, whereas contemporary guidelines increasingly emphasize active surveillance as the preferred management strategy for appropriately selected patients with low-risk prostate cancer. Because the individual clinical and patient-related reasons for proceeding with radical prostatectomy rather than active surveillance were not consistently documented in the retrospective database, the appropriateness of treatment selection for individual low-risk patients cannot be retrospectively determined. This issue should be considered when interpreting the generalizability of the findings.

Finally, substantial biopsy-to-final pathology Grade Group discordance was observed in this cohort. Among the 70 patients with biopsy ISUP Grade Group 1 disease, final pathological Grade Group data were available for 69 patients. Of these, 18 remained Grade Group 1, whereas 51 (73.9%) were upgraded to Grade Group ≥ 2 following radical prostatectomy. Both preoperative prostate biopsy specimens and radical prostatectomy specimens were evaluated by pathologists at the same institution, reducing the likelihood that this discrepancy resulted from inter-institutional variation in pathological interpretation. The observed upgrading may instead reflect the inherent sampling limitations of prostate biopsy, intratumoral heterogeneity, and the more comprehensive histopathological assessment provided by examination of the entire prostatectomy specimen. This relatively high upgrading rate should be considered when interpreting preoperative risk classification and treatment selection in this retrospective cohort.

Overall, the present findings demonstrate that RS-RARP was feasible and associated with acceptable perioperative outcomes and favorable early urinary continence recovery in this single-center cohort. Pathological outcomes, including the observed positive surgical margin rate, were within the range reported in previous RS-RARP series. However, the absence of a comparator group, retrospective design, relatively short oncological follow-up, and incomplete longitudinal PSA and recurrence-related data preclude conclusions regarding comparative or long-term oncological efficacy. Prospective comparative studies incorporating standardized functional assessment, systematic PSA monitoring, and longer oncological follow-up are needed to further define the role of RS-RARP in contemporary prostate cancer surgery.

## 5. Conclusions

In conclusion, this retrospective single-cohort study showed that Retzius-sparing robot-assisted radical prostatectomy was associated with low perioperative morbidity, acceptable pathological outcomes, and favorable early urinary continence recovery. However, the absence of a conventional RARP comparator group, the relatively short follow-up period, and the lack of systematically recorded longitudinal recurrence data preclude conclusions regarding comparative effectiveness, biochemical recurrence-free survival, and long-term oncological efficacy. Prospective multicenter comparative studies with standardized functional assessment and systematic long-term oncological follow-up are warranted to further define the outcomes of RS-RARP.

## Figures and Tables

**Table 1:** Baseline demographic and preoperative characteristics.

Variable	*n* = 132
Age, years	62.0 (57.0–66.0)
BMI, kg/m^2^	26.4 (24.9–28.1)
Charlson Comorbidity Index	1.0 (0.0–1.2)
Diabetes mellitus	15 (11.4)
Hypertension	50 (37.9)
Coronary artery disease	19 (14.4)
PSA, ng/mL	6.45 (4.72–10.15)
Positive biopsy cores	3.5 (2.0–5.0)
ISUP Grade Group ≥ 3	16 (12.1%)
EAU low risk	59 (44.7%)
EAU intermediate risk	60 (45.5%)
EAU high risk	13 (9.8%)

**Table 2:** Perioperative outcomes following Retzius-sparing robot-assisted radical prostatectomy.

Variable	Overall (*n* = 132)
Surgery time (skin-to-skin), min	170.0 (160.0–200.0)
Console time for prostatectomy, min	100.0 (90.0–112.5)
Total console time, min	107.5 (90.0–120.0)
Estimated blood loss, mL	100.0 (70.0–150.0)
Number of lymph nodes removed among patients undergoing lymphadenectomy,	median (IQR)—13 (10–16)
Pelvic lymph node dissection performed	23 (17.4)
Abdominal drain placement	132 (100.0%)
Urinary drain placement	0 (0.0%)
Blood transfusion	1 (0.8%)
Catheter removal, postoperative day	12.0 (10.0–14.0)
Overall perioperative complications	4 (3.0%)
Clavien–Dindo grade I	2 (1.5%)
Clavien–Dindo grade II	1 (0.8%)
Clavien–Dindo grade ≥ III	0 (0.0%)

**Table 3:** Pathological outcomes following Retzius-sparing robot-assisted radical prostatectomy.

Variable	Overall (*n* = 132)
Prostate specimen weight, g	52.0 (43.0–60.0)
Final ISUP Grade Group *	
Grade Group 1	18 (13.7%)
Grade Group 2	85 (64.9%)
Grade Group 3	19 (14.5%)
Grade Group 4	4 (3.1%)
Grade Group 5	5 (3.8%)
Pathological T stage	
pT2	59 (45.0%)
pT3a	50 (38.2%)
pT3b	22 (16.8%)
Positive surgical margins	44 (33.6%) ^†^
Capsular involvement	70 (53.4%)
Seminal vesicle invasion	17 (13.1%) ^‡^
Bladder neck invasion	4 (3.1%)
Perineural invasion	131 (100.0%)
Lymph node metastasis (pN-positive)	21 (16.0%) ^§^

* Final ISUP Grade Group data were available for 131 of 132 patients (^†^ Data available for 131 patients. ^‡^ Data available for 130 patients. ^§^ Percentage calculated out of the 131 patients with available pathological data, though pelvic lymph node dissection was performed in only 23 patients.). Percentages were calculated using the available cases (*n* = 131).

**Table 4:** Functional outcomes following Retzius-sparing robot-assisted radical prostatectomy.

Variable	Overall (*n* = 132)
Time to social continence (0–1 pad), days	30.0 (15.0–56.3)
Time to complete continence (0 pad), days	30.0 (15.0–90.0)
Current pad use, *n* = 130	
0 pad/day	108 (82.4%)
1 pad/day	13 (9.9%)
≥2 pads/day	9 (6.9%)
Current continence status, *n* = 130 *	
Continence code 0	108 (82.4%)
Continence code 1	13 (9.9%)
Continence code 2	9 (6.9%)
Erectile rehabilitation performed, *n* = 131	117 (89.3%)
Current erectile function, *n* = 131 ^†^	
Code 0	7 (5.3%)
Code 1	13 (9.9%)
Code 2	1 (0.8%)
Code 3	89 (67.9%)
Code 4	20 (15.3%)
Code 5	1 (0.8%)

* Continence codes: 0 = 0 pads/day; 1 = 1 pad/day; 2 = ≥2 pads/day. ^†^ Erectile function codes based on [insert scoring system, e.g., IIEF-5 questionnaire]: 0 = [definition], 1 = [definition], 2 = [definition], 3 = [definition], 4 = [definition], 5 = [definition].

**Table 5:** Oncological outcomes following Retzius-sparing robot-assisted radical prostatectomy.

Variable	Value
Follow-up duration, months, median (IQR)	12.0 (12–18)
Postoperative PSA available, *n*/*N* (%)	79/131 (60.3)
Postoperative PSA, ng/mL, median (IQR)	0.00 (0.00–0.00)
Last PSA available, *n*/*N* (%)	122/131 (93.1)
Last PSA, ng/mL, median (IQR)	0.00 (0.00–0.00)
Adjuvant treatment, *n* (%)	6/131 (4.6)
Salvage treatment, *n* (%)	0/131 (0)
Documented biochemical recurrence, *n*/*N* (%)	0/131 (0)
Metastatic progression	Not available
Cancer-specific mortality	Not available
Overall mortality	Not available

## Data Availability

The data that support the findings of this study are available from the corresponding authors upon reasonable request. The data are not publicly available due to privacy and ethical restrictions related to patient confidentiality.

## Abbreviations

The following abbreviations are used in this manuscript:

RS-RARP	Retzius-sparing robot-assisted radical prostatectomy
RARP	Robot-assisted radical prostatectomy
C-RARP	Conventional robot-assisted radical prostatectomy
PSA	Prostate-specific antigen
PSM	Positive surgical margin
ISUP	International Society of Urological Pathology
EBL	Estimated blood loss
IQR	Interquartile range
IPSS	International Prostate Symptom Score
BMI	Body mass index
ASA	American Society of Anesthesiologists
CI	Confidence interval
OR	Odds ratio
NeuroSAFE	Neurovascular structure-adjacent frozen-section examination
PSMA	Prostate-specific membrane antigen
PET/CT	Positron emission tomography/computed tomography
